# Identification of TTAGGG-binding proteins in *Neurospora crassa*, a fungus with vertebrate-like telomere repeats

**DOI:** 10.1186/s12864-015-2158-0

**Published:** 2015-11-17

**Authors:** Núria Casas-Vila, Marion Scheibe, Anja Freiwald, Dennis Kappei, Falk Butter

**Affiliations:** Quantitative Proteomics, Institute of Molecular Biology (IMB) gGmbH, Ackermannweg 4, 55128 Mainz, Germany; Cancer Science Institute of Singapore, National University of Singapore, 14 Medical Drive, 117599 Singapore, Singapore

**Keywords:** Interactomics, Mass spectrometry, Telomere, Telosome, Evolution

## Abstract

**Background:**

To date, telomere research in fungi has mainly focused on *Saccharomyces cerevisiae* and *Schizosaccharomyces pombe*, despite the fact that both yeasts have degenerated telomeric repeats in contrast to the canonical TTAGGG motif found in vertebrates and also several other fungi.

**Results:**

Using label-free quantitative proteomics, we here investigate the telosome of *Neurospora crassa*, a fungus with canonical telomeric repeats. We show that at least six of the candidates detected in our screen are direct TTAGGG-repeat binding proteins. While three of the direct interactors (NCU03416 [ncTbf1], NCU01991 [ncTbf2] and NCU02182 [ncTay1]) feature the known myb/homeobox DNA interaction domain also found in the vertebrate telomeric factors, we additionally show that a zinc-finger protein (NCU07846) and two proteins without any annotated DNA-binding domain (NCU02644 and NCU05718) are also direct double-strand TTAGGG binders. We further find two single-strand binders (NCU02404 [ncGbp2] and NCU07735 [ncTcg1]).

**Conclusion:**

By quantitative label-free interactomics we identify TTAGGG-binding proteins in *Neurospora crassa*, suggesting candidates for telomeric factors that are supported by phylogenomic comparison with yeast species. Intriguingly, homologs in yeast species with degenerated telomeric repeats are also TTAGGG-binding proteins, e.g. in *S. cerevisiae* Tbf1 recognizes the TTAGGG motif found in its subtelomeres. However, there is also a subset of proteins that is not conserved. While a rudimentary core TTAGGG-recognition machinery may be conserved across yeast species, our data suggests Neurospora as an emerging model organism with unique features.

**Electronic supplementary material:**

The online version of this article (doi:10.1186/s12864-015-2158-0) contains supplementary material, which is available to authorized users.

## Background

Linear chromosome ends of nearly all eukaryotes consist of repetitive telomeric sequences that protect the integrity of the genome. This function is assisted by interacting proteins, generally referred to as the telosome, which are essential for functional telomeres [1]. In mammals, a protein complex consisting of six core members (TRF1, TRF2, RAP1, TIN2, TPP1 and POT1) protects the TTAGGG-repeat telomeres from recognition by the DNA damage repair machinery [2, 3] with TRF1 and TRF2 being direct double-strand telomere binding proteins and POT1 binding the TTAGGG single-strand overhang. Additionally, HOT1 was recently identified as a third direct double-strand telomere binding protein in mammals involved in telomerase-mediated telomere homeostasis [4].

While baker’s yeast (*S. cerevisiae*) and fission yeast (*S. pombe*) telomeres have been extensively studied and used as a model in telomere biology, they are both rather exceptional, as they feature degenerated telomeric repeats in juxtaposition to canonical repeat motifs found in most other eukaryotes. Furthermore, the telosomes of these two yeast model species differ greatly: In baker’s yeast, the degenerated double-strand repeats are recognized by Rap1 which interacts with Rif1 and Rif2 [5, 6], while in fission yeast the direct double-strand binding protein Taz1 [7] anchors a telomeric multi-protein complex consisting of Taz1, Rap1, Poz1, Ccq1, Tpz1 and Pot1 [1].

Despite their potentially distinct telomere biology, considerable effort has been made to identify telomere binding proteins in these yeast species. While the identification of telomere binding proteins by their ability to bind telomeric repeats in purification experiments seems straightforward, this strategy has become even more applicable recently due to advances in protein identification by mass spectrometry. For instance, proteomic isolation of chromatin fragments (PICh), a reverse ChIP method, has been established on human telomeres [8]. Similarly, classical DNA affinity purification was used to identify TRF1 by mass spectrometry [9] and the same technique in combination with SILAC has uncovered HOT1 as a direct telomeric DNA binding protein [4]. The latter comparison also highlights how advances in proteomics have enabled the discovery of novel telomeric factors. Additionally, a quantitative telomeric chromatin isolation protocol (Q-TIP) based on immunopurification of TRF2 has been used to identify telomeric proteins [10]. However, the known telomere binders in fungi have been identified by different means prior to streamlined applicability of proteomics. Rap1 was first identified based on its recombinant protein being similarly sensitive to protease digestion as the Telomere Binding Activity in *S. cerevisiae* and on both sharing similar size and specificity [11]. Similarly, Taz1 has not been initially identified by its ability to bind telomeric repeats, but as a factor strongly enhancing telomere length in a genetic screen [7]. More recently, a double-strand telomere binding protein in *Yarrowia lipolytica* was identified by sequence comparison focusing on its two myb domains similar to the human TRFs [12]. We reasoned that an unbiased, proteome-wide interactomics approach would thus be beneficial to uncover the telosome in a not yet characterized fungus, as it allows the detection of telomeric proteins without assumptions and/or prior knowledge. We have previously demonstrated that we are able to identify direct telomere binding proteins in mouse and human [4] and here we apply single-step DNA affinity purification combined with quantitative proteomics to identify the direct telomere binding proteins in *Neurospora crassa*.

We chose Neurospora because little is known about the telosome of TTAGGG-repeat telomeric fungi, and it would be appealing to have a model organism that more directly relates to human telomeres in terms of sequence. Indeed, it was recently shown that while fungal and vertebrate telomerase RNAs share a common ancestral core, the *N. crassa* telomerase is more closely related to vertebrates [13]. It is thus likely that its telomere binding proteins are more closely related to those of vertebrate species compared to the current yeast models. Here we investigated a comprehensive telosome for *N. crassa* and uncover proteins directly binding to TTAGGG repeats, providing a basis for further functional characterization of *N. crassa* telomere biology as an emerging model organism.

## Results and discussion

### Identification of the Neurospora telosome by quantitative proteomics

**Fig. 1:**
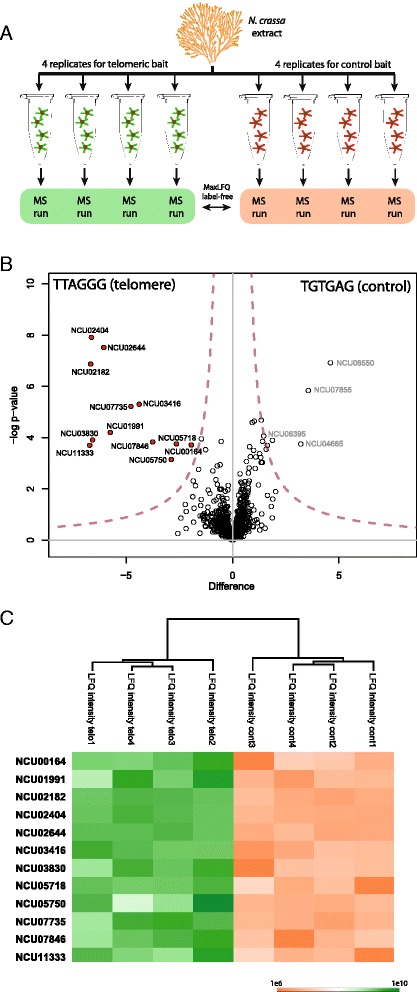
Experimental design and results of Neurospora telomere pull-down. **a** Schematic workflow of the label-free telomere interaction screen. Extracts of *N. crassa* were incubated with either a telomeric probe (TTAGGG) or a shuffled control sequence (TGTGAG). For each probe, four pull-downs were performed independently and measured using high resolution mass spectrometry. To identify TTAGGG repeat binding proteins the MaxLFQ algorithm was used to compare individual peptide intensities between the telomeric bait and the shuffled control sequence. **b** Volcano plot of the telosome of *N. crassa*. We identified 12 proteins (two-sided *t*-test, Welch, FDR = 0.05) to be enriched at the telomeric TTAGGG probe compared to the shuffled control. **c** Heatmap representation of z-scored enrichment values obtained from MaxLFQ intensities for the 12 significantly enriched Neurospora proteins validates reproducible binding behaviour in each replicate

For the identification of telomere binding proteins we performed telomere pull-downs [4] with cellular extracts from *N. crassa* in quadruplicates for a telomeric (TTAGGG) and a control (TGTGAG) bait (Fig. 1a). The bound proteins were eluted from the immobilized oligonucleotides by boiling and separated by one-dimensional gel electrophoresis. Each sample lane was cut into four slices to reduce sample complexity for subsequent analysis. The gel pieces were digested with trypsin and peptides measured on a mass spectrometer. After measurement of each individual pull-down, we employed label-free quantitative analysis empowered by MaxLFQ [14] to obtain enrichment values between target and control bait. We only considered proteins that were detected and quantified in three of our four replicates introducing a very stringent reproducibility filter. By this approach, we identified and quantified 709 proteins of which only 12 were significantly (FDR = 0.05) enriched at the telomeric TTAGGG repeat (Fig. 1b, Additional file 1: Table S1). Each of these 12 proteins shows strong enrichment at the telomere versus control bait in all replicates (Fig. 1c). For the control probe, we used a shuffled TTAGGG sequence also employed in our previous analysis in human and mouse [4]. This control sequence was chosen to ensure that neither the repetitive nature of the telomeric motif nor the exact base content would present a bias in hit selection. While this sequence constitutes an artificial repeat not present in Neurospora, we detected 4 uncharacterized proteins (Fig. 1b) that associate with this TGTGAG repeat motif.

### Evolutionary conservation of TTAGGG-associated proteins in fungi

**Fig. 2:**
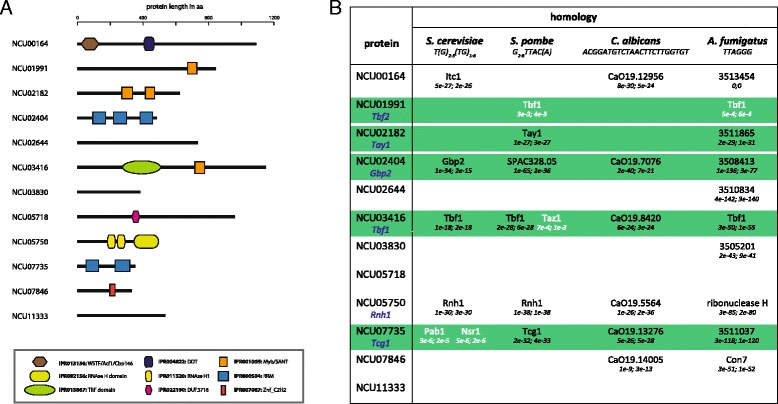
Structure and evolutionary conservation of TTAGGG-associated proteins. **a** Domain structure of Neurospora proteins reported by InterPro 46.0 (http://www.ebi.ac.uk/interpro/). The position of the domains and length of the proteins are drawn to scale. **b** Overview of the corresponding homologs in *S. cerevisiae*, *S. pombe*, *C. albicans* and *A. fumigatus* as determined by sequence similarity search. The telomeric motifs are indicated for each species. Blast hits in black are true homologs whereas blast hits in white showed weak homology and the reverse blast identified a different homologous protein. The first e-value represents the search of the *N. crassa* protein vs. the respective species and the second e-value the reverse blast search. *N. crassa* names in blue are proposed based on experimental data from this study

Upon inspection of the 12 TTAGGG-enriched proteins, we noticed that half of them (NCU01991, NCU02182, NCU02404, NCU03416, NCU07735 and NCU07846) had an annotated nucleic acid binding domain (Fig. 2a). However, as protein annotations and functional descriptions of proteins in Neurospora are extremely limited, we used sequence similarity comparisons with other yeasts to determine the homologs of our candidates in order to obtain further information (Fig. 2b). To cover homology in all major yeast branches, we performed reverse blast searches against *S. cerevisiae*, *S. pombe* and *Candida albicans*. We further included the fungus *A. fumigatus* in our analysis, which also has a TTAGGG-repeat telomere and is more closely related to *N. crassa*.

As expected, we obtained the highest number of homologs (10 of 12) in the closely related fungus *A. fumigatus.* For around half of our candidates, we were still able to also identify homologs in the other three yeast species. While the proteins from *C. albicans* and *A. fumigatus* are not characterized, nearly all homologs in *S. cerevisiae* and *S. pombe* were already implicated in (sub)-telomere regulation, confirming that we indeed identified telomeric proteins by our quantitative mass spectrometry screen (Fig. 1b). NCU07735 is orthologous to the single-strand binding protein Tcg1 from *S. pombe* [15] and also has a weak homology to the telomeric ssDNA-binding protein Nsr1 of *S. cerevisiae* [16]. We equally identified the homolog of the *S. cerevisiae* telomeric ssDNA-binding protein Gbp2 [16] called NCU02404. Cross-confirming this finding, we also affinity-purified Gbp2 from a similar telomere pull-down screen using a degenerated telomeric repeat with *S. cerevisiae* extracts (unpublished data). NCU03416 is the homolog of the essential Tbf1 (TTAGGG-binding protein 1) in *S. cerevisiae* [17]. Furthermore, NCU02182 is homologous to Tay1 [12], which can bind both the *Yarrowia lipolytica* telomeric sequence (GGGTTAGTCA) as well as TTAGGG repeats [18]. Finally, NCU01991 has weak homology to Tbf1 and with the presence of a myb/homeobox domain (Fig. 2a) shows the common DNA-binding domain of telomeric proteins. To use comparable naming to yeast, we suggest to rename NCU03416 as Tbf1, NCU01991 as Tbf2, NCU02182 as Tay1, NCU02404 as Gbp2 and NCU07735 as Tcg1 in Neurospora.

### Investigation of binding characteristics of the Neurospora proteins

**Fig. 3:**
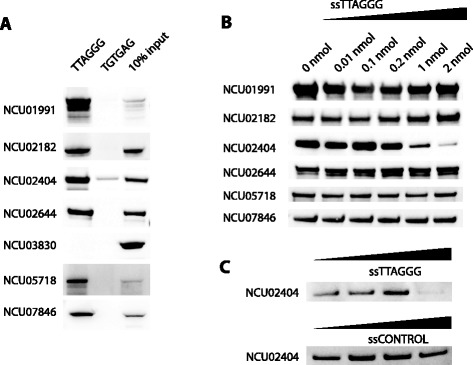
Characterization of binding mode of Neurospora TTAGGG-binding proteins. **a** Western blots from pull-downs of recombinantly expressed Neurospora proteins incubated with concatemerized oligonucleotides of telomeric repeats (TTAGGG) or a shuffled control sequence (TGTGAG) to check for direct binding. **b** Comparative binding test for double-stranded versus single-stranded telomeric DNA. Direct TTAGGG-binding proteins from (**a**) were incubated with increasing amounts of single-stranded TTAGGG repeats. Only NCU2404 binding is decreased at higher ssTTAGGG oligonucleotide concentrations revealing the other 5 proteins as double-strand binders. **c** Western blot of the competition assay of NCU02404 shows that its binding can only be competed with ssTTAGGG but not with an unrelated control sequence

As direct telomere binders usually represent key proteins in a telosome, we wanted to identify which of our candidates are direct and specific TTAGGG-repeat binding proteins. For seven candidates we were able to clone the entire coding sequence from cDNA and to express recombinant proteins. Several candidates failed at the cloning step, putatively due to incorrect gene predictions. For instance, during the cloning of NCU05718, we noted a different exon structure compared to its bioinformatics prediction (Additional file 1: Table S2). The seven expressed proteins were tested for their ability to directly bind to our telomeric bait. Strikingly, six of the investigated seven proteins were able to bind directly to the TTAGGG repeat sequences (Fig. 3a). As our bait preparation relies on concatemerization of oligonucleotides with repetitive sequences to longer fragments, our bait carries a short single-stranded overhang. Therefore, we next dissected whether the direct association was due to binding of single- or double-stranded DNA by a competition assay (Fig. 3b,c). Only NCU02404/ncGbp2, which is homologous to the telomeric single-strand binding protein Gbp2 in *S. cerevisiae* [16], showed diminished binding at elevated single-strand TTAGGG competitor concentrations. Binding of NCU02404/ncGbp2 is probably mediated via its RRM domains (Fig. 2a) similar to the binding of several human RRM domain proteins such as hnRNPA1 and hnRNPA2/B1 to telomeric ssDNA [19, 20]. The other RRM-domain protein (NCU07735/ncTcg1) identified as putative telosome member by our screen (but failed during the cloning procedure) is orthologous to the single-strand binding protein Tcg1 from *S. pombe*, illustrating that *N. crassa* may have two single strand telomeric proteins. The other five candidates were validated as double-strand telomere repeat binding proteins. Two of them (NCU01991/ncTbf2 and NCU02182/ncTay1) feature a classical myb/homeobox domain that is also found in the three direct binding vertebrate telomeric factors TRF1, TRF2 and HOT1 [4, 21]. In addition, for NCU07846 the zinc-finger is the only annotated DNA-binding domain in this protein (Fig. 2a), suggesting that it recognizes TTAGGG repeats via this domain. For the last two proteins (NCU02644 and NCU05718) directly recognizing double-stranded TTAGGG repeats in our assay, the DNA binding domain still needs to be identified. For the other four candidates of our proteomic screen, the binding behaviour of NCU00164, NCU05750 and NCU11333 remains to be established, while NCU03830 likely associates via protein-protein interactions. In addition, NCU03146/ncTbf1, one of the proteins that failed during the cloning step, is the direct homolog of the TTAGGG-repeat binding protein Tbf1 and it is therefore also likely a direct telomere binding protein in *N. crassa*. In summary, we propose that the telosome of *N. crassa* contains at least eight direct telomere binding proteins, inferred by direct binding tests and/or homology search.

### Comparison of telomere associated proteins in fungi

**Fig. 4:**
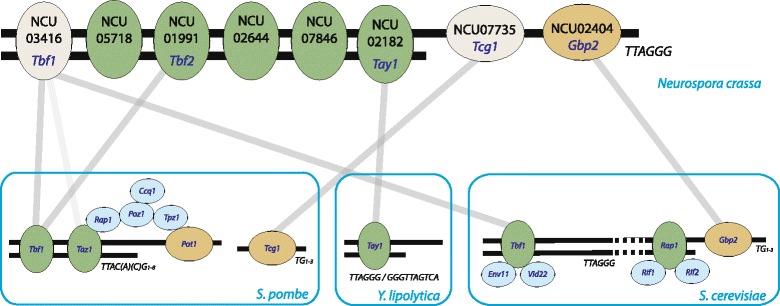
Model for the *N. crassa* telosome based on our mass spectrometric screen and the binding analysis. Neurospora has at least six double strand binding proteins (five were validated experimentally [*green*] and one was inferred from homology [*grey*]) and two single-strand binding proteins of which we have experimentally validated one [*yellow*] while the other [*grey*] was inferred from homology. Connections indicate homologs between Neurospora and the other yeast species

Our quantitative proteomic screen has identified 12 TTAGGG-associated proteins in Neurospora, an organism where no telomeric protein was previously known. Of this set, we classified eight proteins as TTAGGG-repeat binding either inferred from homology and/or validated experimentally (Fig. 4) with two proteins recognizing the single-strand overhang and six proteins binding the double-strand TTAGGG repeat. The single-strand binders have clear homologs in other yeasts, arguing that they are likely functionally conserved. Among the proteins we could not further characterize, the orthologue of NCU05750 (RNase H) has already been shown to be important for RNA-DNA hybrid resolution at *S. cerevisiae* telomeres [22], suggesting that we identified a comprehensive set of telomeric proteins in *N. crassa*.

The identification of a minimum of six TTAGGG double-strand binding proteins in Neurospora is unexpected. When we compare to TTAGGG binding proteins in the closely related telomere models only one protein (Tbf1) has been reported in *S. cerevisiae* and three (Tbf1, Tay1 and Taz1) in *S. pombe* / *Y. lipolytica*. Intriguingly, these three conserved proteins between *S. pombe* / *Y. lipolytica* and Neurospora all contain the classical myb/homeobox domain found also in the vertebrate telomere proteins TRF1, TRF2 and HOT1. While myb/homeobox domain proteins may be linked to core functions at the telomere, additional proteins may have been recruited in evolution to recognize TTAGGG repeats in a context in which they served as actual chromosome extremities. For instance, we here demonstrate that the zinc finger protein NCU07846 can also recognize these repeats and may represent an emerging class of telomere binding proteins, including ZSCAN4 [23] and Ring1b [24]. NCU07846 is the first zinc finger protein shown to directly bind TTAGGG repeats. The high number of TTAGGG-binding proteins in Neurospora may directly relate to the TTAGGG repeat being its telomeric sequence, requiring additional functionality in comparison to *S. cerevisiae* where the TTAGGG sequence is found in the subtelomere. It is furthermore possible that also in *S. pombe* and *S. cerevisiae* not all direct telomere binding proteins are yet known as similar systematic investigations based on affinity purification have not yet been reported for these yeast models.

We did not identify any homologs to the proteins directly binding to the degenerated telomeric repeats in *S. cerevisiae* and *S. pombe* in our screen. This might be the result of divergent evolution and it is therefore attractive to speculate that the degenerated repeat telomeres and their binding proteins are a specialization in these yeast species and that they co-exist with a rudimentary core TTAGGG-repeat binding machinery shared among all yeast species. Based on our knowledge of Neurospora telomere binding proteins, it is likely that Tbf1 end-capping in *S. cerevisiae* [25] is a remnant of an evolutionarily retained function. The additional TTAGGG- associated proteins Reb1, Env11, Vid22 in *S. cerevisiae* [26–28] likely reflect adaptations to the use of TTAGGG repeats as *S. cerevisiae* subtelomeres. It seems therefore likely that the canonical repeats such as TTAGGG are the actual ancestral core found in the vast majority of species in all clades and that the degenerated repeats in *S. cerevisiae* and *S. pombe* are a special telomeric situation in some fungi. Our data strongly supports the hypothesis that Tbf1 homologs in fungi originate from a common ancestor with a function in telomere regulation [29].

## Conclusion

Our current experimental data sheds light on the evolutionary conservation of telomere-binding proteins and uncovers more TTAGGG-binding proteins in Neurospora as expected, suggesting it as an attractive new model species in telomere biology.

## Methods

### Preparation of Neurospora extracts

*Neurospora crassa* strain 74-OR23-1VA biomass was resuspended in PBB buffer (150 mM NaCl, 50 mM Tris–HCl pH 8.0, 10 mM MgCl_2_, 0.5 % Igepal CA630, 2 mM EDTA) and shock frozen in liquid nitrogen. The pellets were lysed by bead milling (MS400, Retsch) in a 25 ml stainless steel container and a steel ball at 30 Hz three times for 5 min. The sample was cooled in liquid nitrogen during the breaks. The frozen biomass was transferred and the melted sample was centrifuged for 1 min at 13,000 g. The supernatant was aliquoted and stored at −80 °C until usage. Protein concentration was determined by Bradford assay (Biorad).

### Telomere pull-down

The biotinylated (TTAGGG) and (TGTGAG) baits were prepared as previously described [4]. Either 1 mg of Neurospora extract (MS experiment) or 200 μg of *E. coli* lysate containing recombinant protein (Western blot) were incubated with 500 μg Dynabeads Streptavidin (Life Technologies) coupled with either telomeric or control DNA for 1 h at 4 °C in PBB buffer (150 mM NaCl, 50 mM Tris–HCl pH 8.0, 10 mM MgCl_2_, 0.5 % Igepal CA630, Complete Protease Inhibitor without EDTA (Roche)) under slight agitation. In the case of the competition experiment increasing amounts (0 nmol, 0.01 nmols, 0.1 nmols, 0.2 nmols, 1 nmols, 2 nmols) of single-strand competitor (TTAGGG)_10_ or a non-specific 57 nt single-strand control oligonucleotide were added to the incubation. After three washing steps with PBB buffer, bound proteins were boiled in 1x LDS buffer containing 100 mM DTT (Life Technologies) at 70 °C and separated on NuPAGE Novex Bis-Tris precast gels (Life Technologies).

### MS sample preparation

Each gel lane was cut into four slices and each slice was minced prior to transfer to an Eppendorf tube. The samples were destained in 50 % ethanol/25 mM ammonium bicarbonate pH 8.0 buffer twice for 30 min, reduced with 10 mM DTT at 56 °C and alkylated with 55 mM iodoacetamide in the dark. The gel pieces were incubated with MS-compatible trypsin (Promega) at 37 °C overnight. Peptides were eluted from the gel pieces using pure acetonitrile and extraction buffer (70 % acetonitrile/30 % ammoniumbicarbonate buffer pH 8/0.1 % TFA). After removal of the acetonitrile in a concentrator (Eppendorf) the peptides were loaded onto a C18 StageTip [30] to be stored.

### Mass spectrometry measurement

The peptides in each fraction were separated by C18 reversed phase chromatography. The separation capillary was packed with Reprosil-Pur 120 C18-AQ, 3 μm (Dr. Maisch). The peptides were separated on a 145 min gradient from 5 to 40 % acetonitrile in 0.1 % formic acid using an EasyHPLC (Proxeon) coupled online to a LTQ Orbitrap XL mass spectrometer (Thermo Scientific). The mass spectrometer was operated in data-dependent acquisition mode performing top10 MS/MS (CID) per full scan (60,000 resolution, scan window 350–1750 m/z) controlled by the Xcalibur software.

### Data analysis

The raw data was processed with MaxQuant [31] 1.4.0.8 using the Neurospora version 3 database (9873 entries) with standard settings except the following (FastLFQ was deactivated and match between runs was activated). The results including LFQ (label-free quantitation) intensities were loaded into Perseus (version 1.4.0.6). Protein groups consisting of only identified by site proteins, reverse hits and contaminants were removed. Proteins that were detected in three of the four TTAGGG replicate measurements were considered. Missing values were imputed in each column separately by using a normal distribution with a width of 0.3 downshifted by 1.8 (standard values). To obtain the LFQ enrichment, the difference of the arithmetic mean of the quadruplicates of control and bait was calculated and plotted (x-axis). A two-sided *t*-test with 250 randomizations was performed between the four replicates of the telomeric bait against the four replicates of the control baits. A significance threshold was determined at an FDR of 0.05 and s_0_ = 0.2. The results were exported and plotted in R (version 2.0.8.1).

### BLAST and evolutionary analysis

Annotated *N. crassa* peptide sequences of the 12 hits were blasted using the NCBI blastp (protein-protein BLAST) standard search. Searches were carried out against *S. cerevisiae* (taxid:4932), *S. pombe* (taxid:4896), *A. fumigatus* (taxid:746128) and *C. albicans* (taxid:5476). Blast hits were reverse blasted against *N. crassa* (taxid:5141) as search organism to verify homology. Homologs were only considered valid if both the forward and reverse blast yielded e-values smaller than e-8 and if in both directions the respective proteins were each the strongest hit. In some cases also weak homologs (e-values smaller than e-3) are reported, in which the forward and/or reverse blast identified a different protein as the direct homolog.

### Cloning of Neurospora transcripts

Total RNA was extracted from *N. crassa* 74-OR23-1VA strain cells using the PrepEase RNA spin kit (USB) according to the manufacturer’s yeast protocol. The RNA was reverse transcribed using the protocol for polyadenylated mRNA of the First strand cDNA synthesis kit (Thermo). The transcripts of interest were amplified using chemically synthesized oligonucleotides (Metabion, Additional file 1: Table S3) and were cloned into pCR8-TOPO (Life Technologies) using TA cloning. Inserts were validated by Sanger sequencing prior to transfer into the pCoofy4 (N-terminal His-MBP-tag) expression vector using SLIC cloning [32]. In cases were amplified sequences did not match to the Neurospora transcript prediction, a complete procedure was repeated starting from total RNA extraction of Neurospora cells.

### Recombinant protein expression

The pCoofy4 expression vectors were transformed by heat-shock into *E. coli* BL21. Bacteria were grown overnight at 25 °C in auto-induction media [33] and harvested at an OD600 8–12 by centrifugation at 3000 g for 10 min at 4 °C. Cell pellets were resuspended in lysis buffer (100 mM NaCl, 50 mM Tris–HCl pH7.5, 10 mM MgCl_2_) to an OD600 of 6 and lysed by bead milling using 0.1 mm diameter Zirconia/glass-beads (Carl-Roth) in a tissue lyzer (Precellys) twice at 5600 rpm for 30 s. Cell debris was removed by centrifugation and protein concentration was determined by Bradford assay (Biorad). Successful expression and solubility of recombinant proteins were assessed by SDS-PAGE. Lysates were stored at −20 °C in 10 % glycerol.

### Western blot

Proteins were transferred to a Protran 85 nitrocellulose membrane (GE Healthcare) using a wet blot system in transfer buffer (20 % methanol, 25 mM Tris–HCl pH7.5, 150 mM glycine) at 300 mA for at least 90 min. The membrane was blocked and incubated with the Penta-His HRP conjugate kit (Qiagen) according to the manufacturer’s instructions. Individual bands were detected using ECL Detection Reagent (GE Healthcare) and visualized by a Biorad ChemiDoc System.

## Supporting data

The data set supporting the results of this article is included within the article and its additional files.

## Additional file

Additional file 1: Table S1. MaxQuant output table containing protein identification and LFQ quantification data for the neurospora telomere pull-downs. **Table S2.** Sequence of the cloned coding sequences from *N. crassa* by cDNA synthesis of its mRNA. **Table S3.** Primers used in this study. (XLSX 126 kb)
